# Sepsis-Associated Disseminated Intravascular Coagulation and Thromboembolic Disease

**DOI:** 10.4084/MJHID.2010.024

**Published:** 2010-08-13

**Authors:** Nicola Semeraro, Concetta T. Ammollo, Fabrizio Semeraro, Mario Colucci

**Affiliations:** Department of Biomedical Sciences and Human Oncology, Section of General and Experimental Pathology, University of Bari, Bari, Italy

## Abstract

Sepsis is almost invariably associated with haemostatic abnormalities ranging from subclinical activation of blood coagulation (hypercoagulability), which may contribute to localized venous thromboembolism, to acute disseminated intravascular coagulation (DIC), characterized by massive thrombin formation and widespread microvascular thrombosis, partly responsible of the multiple organ dysfunction syndrome (MODS), and subsequent consumption of platelets and coagulation proteins causing, in most severe cases, bleeding manifestations. There is general agreement that the key event underlying this life-threatening sepsis complication is the overwhelming inflammatory host response to the infectious agent leading to the overexpression of inflammatory mediators. Mechanistically, the latter, together with the micro-organism and its derivatives, causes DIC by 1) up-regulation of procoagulant molecules, primarily tissue factor (TF), which is produced mainly by stimulated monocytes-macrophages and by specific cells in target tissues; 2) impairment of physiological anticoagulant pathways (antithrombin, protein C pathway, tissue factor pathway inhibitor), which is orchestrated mainly by dysfunctional endothelial cells (ECs); and 3) suppression of fibrinolysis due to increased plasminogen activator inhibitor-1 (PAI-1) by ECs and likely also to thrombin-mediated activation of thrombin-activatable fibrinolysis inhibitor (TAFI). Notably, clotting enzymes non only lead to microvascular thrombosis but can also elicit cellular responses that amplify the inflammatory reactions. Inflammatory mediators can also cause, directly or indirectly, cell apoptosis or necrosis and recent evidence indicates that products released from dead cells, such as nuclear proteins (particularly extracellular histones), are able to propagate further inflammation, coagulation, cell death and MODS. These insights into the pathogenetic mechanisms of DIC and MODS may have important implications for the development of new therapeutic agents that could be potentially useful particularly for the management of severe sepsis.

## Introduction:

Sepsis is a serious and relatively common disorder and represents the leading cause of mortality in non-coronary intensive care units worldwide. Sepsis is almost invariably associated with haemostatic abnormalities ranging from isolated thrombocytopenia and/or subclinical activation of blood coagulation (hypercoagulability), to sustained systemic clotting activation with massive thrombin and fibrin formation and subsequent consumption of platelets and proteins of the haemostatic system (acute disseminated intravascular coagulation, DIC).^1^ From a clinical standpoint, isolated thrombocytopenia, which is seen mainly in viral infections, is only occasionally serious enough to cause a bleeding diathesis. Although it may be immune mediated, other non immune pathogenetic mechanisms might be involved, including decreased thrombopoiesis, direct interaction of the virus with platelets and increased sequestration by the spleen or at the endothelial level due, for instance, to virus-induced endothelial injury.^2^ Septic patients may also present with localized thrombotic manifestations. Several studies, indeed, have shown that patients with severe infectious diseases are at increased risk for venous thrombosis and pulmonary embolism.^3^–^5^ The most common and dramatic clinical feature of sepsis-associated DIC, however, is widespread thrombosis in the microcirculation of different organs which may importantly contribute to solitary or multiple organ dysfunction. The development of the multiple organ dysfunction syndrome (MODS) is a major determinant of mortality in sepsis.^1^,^2^,^6^ Therefore, health care providers must be aware of the signs of organ dysfunction and specifically look for the occurrence of this complication. In fulminant DIC, the consumption and subsequent exhaustion of platelets and coagulation proteins will result in simultaneous bleeding of different severity, ranging from oozing at arterial or venous puncture sites to profuse haemorrhage from various sites. DIC is classically associated with Gram negative bacterial infections but it can occur with a similar incidence in Gram positive sepsis. Moreover, systemic infections with other micro-organisms, such as viruses, *Rickettsiae* and even parasites (e.g. *Plasmodium falciparum*) may also result in DIC.^6^

The pathophysiology of sepsis-associated DIC is extremely complex and still represents a matter of extensive investigation. The key event is the systemic inflammatory response to the infectious agent.^7^,^8^ The causative micro-organisms express unique cellular constituents not found in vertebrate animals, now referred to as pathogen-associated molecular patterns (PAMPs), or microbial-associated molecular patterns (for example endotoxin or lipopolysaccharide, LPS). Moreover, during infection, inflammation or stress, host cell-derived factors are generated as “danger signals” (alarmins) which, together with PAMPs, are referred to as danger-associated molecular patterns (DAMPs). These elements are recognized via specific receptors called pattern recognition receptors (PRRs), among which the Toll-like receptors (TLRs) and the complement receptors, by immune and other host cells (monocytes-macrophages, platelets and endothelial cells among others). The subsequent activation of specific intracellular signal-transduction pathways eventually results in the synthesis of a number of proteins including proinflammatory cytokines. The latter, together with other mediators generated by the inflammatory cascade, including those derived from complement activation,^9^ act in concert with the micro-organisms and/or their products to trigger the coagulation pathways, DIC and organ dysfunction.^1^,^10^,^11^ Interestingly, enzymes generated during the clotting cascade can also interact with specific cellular receptors thus eliciting cell responses that amplify the inflammatory reactions.^12^,^13^ Inflammation can also result in cell apoptosis or necrosis^7^ and recent evidence indicates that products released from dead cells, such as nuclear proteins, are able to propagate further inflammation, coagulation, cell death and organ failure.^7^,^14^,^15^ This article summarizes current knowledge on the pathogenesis of DIC and multiple organ dysfunction and the ensuing development of potential therapeutics.

## Pathogenesis of sepsis-associated coagulopathy and thrombus formation

In sepsis the causative agent and the associated inflammatory response drive fibrin formation and deposition by several simultaneously acting mechanisms which include 1) up-regulation of procoagulant pathways, 2) down-regulation of physiological anticoagulants and 3) suppression of fibrinolysis.^1^,^6^,^11^,^16^

### Up-regulation of procoagulant pathways:

The aberrant in vivo expression of TF plays a pivotal role in the activation of blood coagulation in the setting of sepsis and endotoxemia. Indeed the impairment of the TF pathway by various means prevents blood clotting abnormalities (including fibrin deposition in target tissues) and, in some models, also the lethal effects of endotoxemia.^11^,^17^–^21^ Conversely, inhibition of the contact system by anti-factor XII antibodies, while preventing lethal hypotension, does not influence the development of DIC in septic baboo.^22^ The prevention of hypotension, which is most likely mediated by the generation of bradykinin, indicates that activation of the contact system does take place in sepsis and is likely elicited not only by the micro-organism or endotoxin, as originally thought, but also by recently discovered mechanisms including polyphosphate release by activated platelets and/or by bacteria^23^,^24^ and extracellular RNA derived from damaged or necrotic cells.^25^ The implication of TF in sepsis-associated DIC is also supported by the observation that the plasma levels of the protein are increased in septic patients and are generally associated with raised concentrations of markers of clotting activation.^26^–^29^ Moreover, in some studies, a significant correlation was found between TF levels and organ dysfunction.^27^,^29^

**Figure 1.**
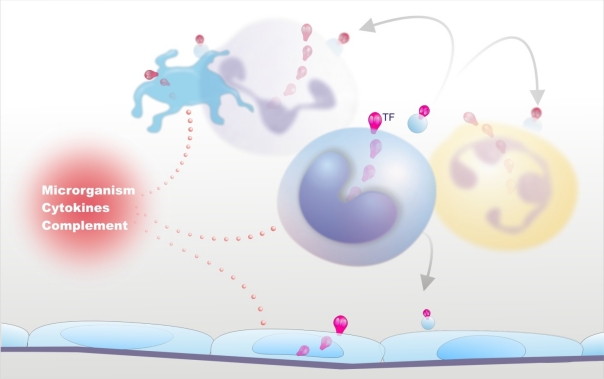
Up-regulation of Tissue Factor (TF) in Sepsis. Infectious agents, their products, and endogenous inflammatory mediators induce TF synthesis in various blood cells as well as the release of TF-bearing microparticles from activated or apoptotic cells. The latter may fuse with different cells and make them capable of activating coagulation. Available evidence point to monocytes-macrophages as the most important procoagulant cells in sepsis.

Despite this evidence, the question remains: what is cellular source of TF in sepsis? In vitro, several cell types that are haemostatically inactive under normal conditions, when exposed to a number of agonists, may undergo profound functional alterations that effectively transform their membrane into a procoagulant surface. The pivotal step causing this shift is the synthesis and/or the expression of TF. Endothelial cells (ECs) and mononuclear phagocytes have been most extensively studied in this respect (Figure 1). These cells can be induced to synthesize TF by a wide variety of stimulating agents or conditions that are of pathophysiological importance in sepsis (Table 1).^10^,^30^–^39^ Of particular relevance are, besides micro-organisms and their products (LPS, the most powerful TF inducer and a major player in Gram negative sepsis; peptidoglycan and lipotheicoic acid, involved in Gram positive sepsis, and several exotoxins), the pro-inflammatory cytokines and chemokines,^10^,^33^–^35^ and the more recently discovered high mobility group box-1 (HMGB-1),^39^ which will be discussed later. It is remarkable that the classical anti-inflammatory cytokines, namely IL-4, IL-10, IL-13 are able to inhibit TF synthesis induced by inflammatory stimuli, providing further support to the close relationship between inflammation and coagulation.^40^ Another important mechanism for TF induction is provided by cellular interactions, including the interaction of ECs or monocytes with T-lymphocytes, natural killer cells, activated platelets and smooth muscle cells (SMCs).^33^–^35^ These cell-cell interactions may occur through different pathways, such as CD40 ligation on ECs and monocytes by CD154 (CD40 ligand)-expressing cells (e.g. T-lymphocytes, platelets, SMCs), binding of P-selectin glycoprotein ligand-1 (PSGL-1) on monocytes to P-selectin on activated platelets or ECs and the production of soluble mediators, including cytokines and unknown factors.^33^–^35^,^41^ The observation that *Plasmodium falciparum*-infected red cells are able to induce TF synthesis in ECs through cell-cell contact provides a possible mechanism for triggering coagulation in DIC associated with parasitic infections. Of note, parasitized red cells also participate in the clotting amplification phase by supporting the assembly of tenase and prothrombinase complexes.^42^

ECs and monocytes may also exhibit other properties promoting coagulation events on their surface, including the production of factor V and of a factor V activator and the expression of specific, though ill defined, membrane receptors for several clotting factors,^33^,^43^ whereby they can propagate the coagulation reactions till thrombin formation. Interestingly, several inflammatory mediators, including TNF, pathogenic micro-organisms, LPS and complement membrane attack complex C5b-9, may amplify the tenase and the prothrombinase assembly on the cell surface, eventually leading to an increased rate of thrombin formation.^33^,^43^ This effect is likely due to the change in membrane asymmetry (exposure of anionic phospholipids, especially phosphatydilserine) required for activation of prothrombin (and factor X). Thrombin formation on the surface of monocytes-macrophages may be further facilitated by some properties unique to these cells. Indeed, monocytes constitutively express at their surface elastase and cathepsin G that activate factor V to Va.^43^ The generated active cofactor remains bound to cell membrane, so that, following factor Xa formation, a functionally efficient prothrombinase complex can be assembled on the cell surface. In addition, factor Va bound to monocytes is protected from inactivation by activated protein C,^44^,^45^ implying that factor Va activity remains sustained on the cell surface and that down-regulation of monocyte prothrombinase is impaired. Because of this complex array of procoagulant properties, perturbed ECs and, especially, activated monocytes-macrophages may provide a surface onto which the clotting pathways are initiated and propagated, eventually leading to fibrin formation in the cell microenvironment. Fibrin, once formed, might function as a modulator of the coagulation response by its ability to down-regulate the TF response of monocytes to LPS or other inflammatory stimuli.^46^

More recent in vitro studies have reported TF expression by other blood cells, namely human polymorphonuclear leukocytes (neutrophils and eosinophils) and platelets (Figure 1). Neutrophils produce and express functional TF in response to inflammatory agents (C5a, bacterial peptide fMLP, P-selectin)^47^,^48^ while eosinophils contain preferentially in their specific granules preformed TF, which is translocated to the cell membrane upon stimulation with platelet activating factor (PAF) or with PAF plus GM-CSF.^49^ Other studies, however, found that neither neutrophils nor eosinophils express TF but can acquire TF by binding monocyte-derived microparticles (MPs).^50^–^52^ Different groups have described the presence of appreciable amounts of TF also in human platelets and its translocation in the functionally active form to the cell surface in response to classical platelet agonists such as collagen, ADP and thrombin.^53^,^54^ As to the origin of platelet TF, quiescent and stimulated platelets have been shown to express variable levels of TF mRNA and protein.^54^,^55^ Moreover, Schwertz et al.^56^ demonstrated that human platelets contain TF pre-mRNA and that upon activation this is spliced into mRNA and translated into protein. Again, however, other groups failed to detect any TF protein or activity on resting or calcium ionophore-stimulated human platelets.^57^,^58^ Therefore, TF expression by human platelets and polymorphonuclear leukocytes remains controversial.

In addition to triggering and propagating blood clotting, ECs, monocytes-macrophages and other cells may support coagulation by yet another mechanism, namely the formation of microparticles (MPs) (Figure 1). Indeed, cell activation by several inflammatory stimuli, as well as cell apoptosis, is accompanied by the formation and release into the extra-cellular space of fragments of their plasma membrane named MPs or microvesicles that retain the properties of the cells of origin, among which TF and anionic phospholipids.^35^,^59^ Interestingly, these MPs can be transferred to the surface of other cells via adhesive receptors (for instance PSGL-1 on leukocyte-derived MPs and P-selectin on activated platelets or ECs) and this interaction results in increased TF activity.^35^,^59^ In this way, MP-bearing cells too become capable of triggering and propagating coagulation.

Although all mentioned cells might contribute to the aberrant in vivo expression of TF, most available studies point to activated monocytes-macrophages as the main triggers of blood coagulation during sepsis or endotoxemia. Indeed, monocytes-macrophages of different origin (blood, spleen, peritoneal, alveolar, marrow) obtained from animals treated with endotoxin or bacteria express strong TF activity.^17^,^33^,^60^–^64^ Studies in different animal models of endotoxemia have shown increased TF expression in important target organs in which fibrin deposition is often observed during DIC, namely lung, kidney, liver, spleen and brain.^17^,^61^,^63^–^65^ At cellular level TF was detected rather frequently in monocytes present in the microcirculation and in macrophages infiltrating the involved tissues^64^–^66^ and, in some studies, also in tissue cells, e.g. alveolar epithelial and alveolar type II cells, tubular and/or glomerular epithelial cells, and in astrocytes.^61^,^63^,^64^,^67^ In addition, a selective genetic deficiency of TF expression by hematopoietic cells as well as the deletion of TF gene in myeloid cells was found to reduce LPS-induced coagulation, inflammation, and mortality in mice.^20^,^68^,^69^ Increased expression of monocyte-macrophage TF has been also documented in human beings, in particular in healthy volunteers after administration of low-dose endotoxin,^70^,^71^ in septic patients and other conditions with associated endotoxemia (e.g. obstructive jaundice),^62^,^72^,^73^ and in patients with peritonitis (in peritoneal macrophages) or acute respiratory distress syndrome (in broncho-alveolar lavage, BAL, fluid).^62^,^74^ Interestingly, in some studies, monocyte TF was related with blood clotting activation, severe organ dysfunction and lethal outcome.^62^,^72^,^73^ Surprisingly, and in contrast with the abundant in vitro evidence, ECs were negative for TF in most animal studies,^61^,^63^,^65^,^67^ with few exceptions.^64^,^75^,^76^ In one study TF expression was found in ECs within the splenic microvasculature of septic baboons but not in ECs of pulmonary vessels.^64^ Others observed the expression of TF activity on intact aortic ECs of endotoxin-treated rabbits.^75^ More recently, Lupu et al.^76^ observed TF protein and activity especially on ECs at branch points of the aorta of septic baboons and suggested that site-dependent endothelial heterogeneity coupled with rheological factors might influence the focal vascular procoagulant response to sepsis. However, the observation that the presence of TF on ECs was restricted to granular structures some of which were also positive for the leukocyte marker PSGL-1 would suggest that leukocyte-derived MPs may deliver TF to activated ECs in vivo.^76^ These discrepancies in the detection of TF expression on ECs in vivo during sepsis and endotoxemia might be due to different causes including the relative sensitivity of the TF assays, the heterogeneous expression of TF possibly linked to vascular bed-specific response and species-specific differences in vascular response to inflammatory stimuli. Recent findings indicate that the deletion of the TF gene in ECs had no significant effect on activation of coagulation in endotoxemic mice.^69^

Another source of tissue factor in vivo might be polymorphonuclear cells, whose inappropriate activation and positioning within the microvasculature strongly contributes to the pathological manifestations of multiple organ failure.^77^ Indeed, TF-positive neutrophils have been observed in the circulation of septic or endotoxemic animals.^76^,^78^ However, de Vaard et al.^79^ showed that TF-positive granulocytes infiltrating organs do not express TF mRNA in a mouse model of endotoxemia suggesting that they can acquire TF by binding monocyte-derived MPs.

Platelets most likely play a pivotal role in the pathogenesis of coagulation abnormalities in sepsis because they can be activated by proinflammatory mediators, such as PAF and thrombin, and, once activated, may amplify thrombin generation by providing the anionic phospholipid surface onto which coagulation reactions occur.^11^ Moreover, by expressing P-selectin on their surface and/or by releasing soluble P-selectin, platelets may enhance the expression of TF on monocytes.^80^ As to the role of platelet TF, a recent study in a mouse model of endotoxemia failed to detect either TF pre-mRNA or RNA in unstimulated or activated mouse platelets. Moreover, deletion of the TF gene in megakaryocytes had no effect on clotting activation in endotoxemic mice.^69^ On the other hand, freshly-isolated platelets from septic patients more frequently expressed mature TF mRNA and had increased levels of TF protein compared to platelets isolated from healthy controls.^69^ However, it is likely that some of the TF protein associated with platelets in septic patients derives from the binding of leukocyte-derived MPs.^69^ Although these data suggest that platelets may participate in the activation of coagulation during sepsis, the direct involvement of platelet TF synthesis remains controversial.

There are additional sources of TF that might contribute to coagulation activation in sepsis, as clearly suggested by the observation that the selective inhibition of TF expressed by non-hematopoietic cells substantially reduces the activation of coagulation in endotoxemic mice.^69^ The precise cellular source of TF in the non-hematopoietic cell populations is unknown. As mentioned above, in endotoxemic and septic animals, TF expression is increased in many organs and cell types.^61^,^63^,^64^,^67^ Therefore, also considering that the role of EC TF remains uncertain, it is likely that TF up-regulation in multiple extravascular cell types in multiple organs contributes to activation of coagulation during sepsis. Moreover, the well known increase in vascular permeability and vascular damage occurring during severe inflammation will allow the exposure of normal extravascular TF to blood.

Overall, at least in the setting of sepsis and endotoxemia, monocytes-macrophages appear to be the main source of TF, together with specific cells in target tissues. Further support for a prominent role of these cells comes from studies on MPs. In endotoxemic mice, levels of MP TF activity were found to correlate with coagulation activation, suggesting the possible use of TF-positive MPs as a biomarker for evaluating the risk of DIC in Gram-negative sepsis.^81^ Patients with meningococcal sepsis had increased numbers of circulating procoagulant MPs derived from various cells with the highest levels detected in a patient with an extremely fulminant course of DIC.^82^ The procoagulant activity of MPs was due to the expression of both TF and phosphatidylserine on their surface and the majority of TF-positive MPs were of monocyte origin (CD14-positive). Interestingly, the formation of TF-positive procoagulant MPs derived from monocytes (CD14-positive) has been also demonstrated in the model of human low-dose endotoxemia.^83^

### Impairment of physiological anticoagulant mechanisms:

**Figure 2.**
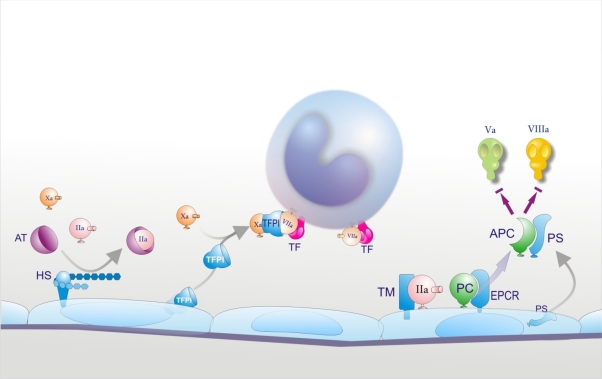
Anticoagulant properties of endothelial cells. Heparan-sulphate (HS) is expressed on the endothelial surface and accelerates the interaction between antithrombin (AT) and its target enzymes, among which factor Xa and thrombin (IIa). Thrombomodulin (TM) and endothelial protein C receptor (EPCR) are also membrane proteins and are involved in protein C (PC) activation. Tissue factor pathway inhibitor (TFPI) and protein S (PS) are released into blood. TFPI, after combining with factor Xa, binds to and neutralizes the TF/VIIa complex on cell surfaces. Protein S is the cofactor of activated protein C (APC) which degrades factors Va and VIIIa. Micro-organisms and inflammatory mediators down-regulate the anticoagulant properties of the endothelium in different ways (see text).

Under physiological conditions EC surface expresses various components involved in the anticoagulant pathways, including thrombomodulin (TM), endothelial protein C receptor (EPCR), protein S, tissue factor pathway inhibitor (TFPI) and the heparin like proteoglycan heparan sulphate (Figure 2).^33^,^84^ EPCR and thrombin bound to TM convert protein C into activated protein C (APC) which is responsible, together with protein S, for the inactivation of critical cofactors Va and VIIIa; TFPI, after binding factor Xa, complexes with TF-VIIa and blocks TF-initiated coagulation; heparan sulphate stimulates the inhibitory activity of antithrombin toward clotting enzymes like thrombin and factor Xa. Because the endothelium plays a critical role in orchestrating the host response to sepsis and it is the target of the pathogen and its products, and of a myriad of host mediators, a number of in vitro and following their interaction with ECs, such as oxidants and/or lysosomal proteases.^33^,^84^ Leukocyte proteases could also contribute to the release of TM from the cell membrane. Inflammatory cytokines may further impair the protein C pathway by reducing protein S secretion and the expression of EPCR in cultured ECs. Moreover, various agents, including cytokines, H_2_O_2_ and thrombin dramatically increase the shedding of EPCR from cells in culture medium.^33^,^84^

At variance with in vitro studies, animal experiments investigating in vivo changes in TM and EPCR produced rather controversial data. While in one study^85^ the infusion of IL-1 in rabbits suppressed TM activity of aortic endothelium, down-regulation of TM could not be observed either in rabbit aorta during experimental endotoxemia or in vascular beds of different organs in two distinct sepsis models in baboons and rats.^64^,^86^ On the other hand, a rise in soluble plasma TM was consistently observed during experimental endotoxemia, suggesting that direct and/or indirect (leukocyte-mediated) endothelial activation-damage in vivo studies evaluated the behavior of endothelial anticoagulant mechanisms in relation to sepsis. Among these, the protein C anticoagulant pathway has been the most extensively studied and, in general, it is down-regulated both in experimental and human sepsis. In vitro, the expression of TM was found consistently reduced in response to most agents listed in Table 1.^33^,^84^

TM reduction has been attributed to different mechanisms including synthesis inhibition, acceleration of internalization and degradation, or inactivation by products of leukocytes released by inflammatory mediators does occur in vivo.^33^,^87^ As to the EPCR, studies in rats and mice showed that EPCR mRNA, rather than falling, as predicted from in vitro experiments with cultured cells, rapidly increased after treatment with lethal doses of LPS.^88^ This was associated with a marked rise in soluble plasma EPCR. The increase of both EPCR mRNA and soluble EPCR was mediated by thrombin, not by TNF.

Although the data summarized above may be difficult to interpret, the importance of the PC anticoagulant pathway for the development of DIC in sepsis is supported by animal experiments showing that compromising the protein C system at different levels (by interfering with PC activation through a monoclonal antibody or by reducing the plasma levels of free protein S with the administration of C4b-binding protein or by treatment with an antibody that blocked the binding of EPCR to PC/APC) resulted in a marked worsening of DIC and in increased morbidity and mortality, whereas restoring an adequate function of APC (by treatment with APC) prevented the coagulopathy and improved survival and organ failure.^1^,^11^,^33^ Interestingly, mice with heterozygous protein C deficiency had more severe DIC and organ dysfunction and a higher mortality than the wild-type controls^89^ and mice homozygous for a point mutation of the TM gene that specifically deletes the anticoagulant activity of the protein (poor binding to thrombin and thus reduced capacity to generate APC), as compared with wild-type mice, exhibited 10- to 30-fold greater amounts of fibrin in the microcirculation of several organs.^90^ In addition, clinical observations indicate that acquired severe protein C deficiency is associated with early death in individuals with sepsis.^91^

Studies in human sepsis have in general confirmed the dysfunction of the protein C pathway. The plasma levels of soluble TM were found to be increased^92^–^94^ and were often correlated with disease severity and poor outcome.^92^,^94^ Interestingly, Saito et al.^95^ found significantly higher TM levels in septic patients with leukocytosis than in those with leukopenia, suggesting an important role for leukocytes in EC perturbation. Patients with sepsis also had increased plasma levels of EPCR,^96^ indicating that the latter can be used as an additional marker of EC activation. Other common findings in septic patients are low levels of PC and PS due to impaired liver synthesis and possibly to consumption (because of degradation by neutrophil elastase), and low levels of free PS, due to increased C4b-binding protein as a consequence of the acute phase reaction.^11^,^33^ Moreover, the expression of TM and EPCR on morphologically intact ECs of dermal vessels was found to be reduced in biopsy specimens of purpuric lesions from children with meningococcal sepsis, as compared with control skin-biopsy specimens.^93^ Plasma levels of APC were low in some of these patients even after treatment with non activated PC concentrates, thus confirming down-regulation of TM in vivo and impaired activation of protein C. Liaw et al.,^97^ using a sensitive assay, observed that plasma levels of APC vary markedly among patients with severe sepsis and, while in some patients they paralleled thrombin generation (assessed by prothrombin F1+2), in others they were low despite elevated F1+2, indicating that APC generation is impaired in the latter. Interestingly, APC plasma levels were significantly higher in survivors than in non survivors (28-day mortality), suggesting that endogenous APC serves protective functions. Indeed, besides exerting anticoagulant and profibrinolytic activity, APC also has important inflammation-modulating effects, including decreased production of inflammatory cytokines and TF by activated monocytes, antioxidant properties, anti-apoptotic activity and prevention of loss of endothelial barrier function.^98^

During sepsis, plasma antithrombin levels are markedly decreased because of consumption resulting from ongoing clotting activation, impaired synthesis and degradation by neutrophil proteases.^11^ The observation that inflammatory stimuli can down-regulate the expression of heparan sulphate proteoglycan in cultured ECs^1^,^33^ suggests an additional mechanism contributing to the reduced antithrombin function in sepsis. Low antithrombin plasma levels have been found to be associated with increased mortality.^6^

With respect to TFPI, in vitro studies evaluating the effect of inflammatory stimuli on the production of this inhibitor by cultured ECs gave conflicting data.^33^ Likewise, controversial reports have been published on the changes of TFPI in plasma during sepsis, ranging from increased levels, probably due to release from ECs, to reduced, likely as a result of consumption, or unchanged levels.^33^ Deeper information on the regulation of TFPI by inflammatory agents have been obtained from animal studies at tissue and cellular level. In rats given endotoxin and in mice given either TNF or endotoxin, a decreased expression of TFPI was found in several organs and in ECs.^65^,^99^ Moreover, Tang et al.^100^ found that lung-associated TFPI antigen and mRNA decreased during *E. Coli*-induced sepsis in baboons, and TFPI activity diminished abruptly as soon as at 2 hours after bacterial challenge. Blocking antibodies against TFPI increased fibrin deposition in septic baboon lungs, suggesting that TF-dependent coagulation might be aggravated by reduced endothelial TFPI. It can be concluded that TFPI under-expression might represent an additional mechanism, together with TF up-regulation, to augment the local procoagulant potential, thus contributing to fibrin formation at tissue level. The importance of TFPI in modulating the procoagulant response to endotoxin in vivo is supported by the observation that, in human experimental low-dose endotoxemia^101^ and in a baboon *E.coli* sepsis model,^18^ the administration of TFPI inhibited thrombin generation and, in the latter model, also reduced the mortality. This effect probably results not only from impaired coagulation but also from the capacity of TFPI to block the cellular effects of endotoxin.^102^

#### Suppression of fibrinolysis:

In sepsis-associated DIC accumulation of fibrin deposits in the microcirculation may be greatly facilitated by an impairment of the fibrinolytic system.^16^,^33^ Infusion of des-A-fibrin or thrombin, at doses unable to induce fibrin accumulation in normal animals, caused diffuse renal microthrombosis in animals pretreated with antifibrinolytic agents. Interestingly, a single endotoxin injection was sufficient to render the animals sensitive to thrombogenic stimuli, most probably because of the inhibition of fibrinolysis. Moreover, administration of high doses of tissue-type plasminogen activator (t-PA) or low doses of plasminogen activator inhibitor-1 (PAI-1)-resistant t-PA prevented fibrin deposition in kidneys of endotoxin-treated rabbits.^33^ Likewise, in a rat model of endotoxemia, fibrin deposition in lungs was decreased by an inhibitor of PAI-1.^33^

**Figure 3.**
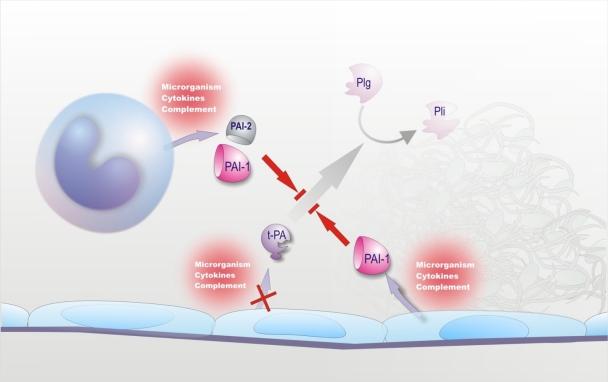
Fibrinolysis suppression in sepsis. Microorganisms, their products, and endogenous inflammatory mediators may lead to hypofibrinolysis by enhancing the release of plasminogen activator inhibitors (mainly PAI-1), particularly by endothelial cells. The same agents may variably influence the release of t-PA (see text for further details).

Endothelium is known to play a pivotal role in the fibrinolytic process through the regulated synthesis and release of key proteins, namely t-PA, urokinase-type PA (u-PA) and PAI-1. The production of these proteins can be modulated in cultured ECs by a number of stimuli or conditions.^33^ Among the agents involved in sepsis-associated DIC, some, such as TNF, IL-1, LPS and herpes simplex virus, had no effect or decreased t-PA synthesis (Figure 3), while others, such as thrombin and factor Xa, increased t-PA production.^33^ However, most of the above stimuli, including those that augmented t-PA release, as well as many others listed in Table 1, consistently stimulated PAI-1 synthesis (Figure 3), the net effect being definitely antifibrinolytic.^33^ It should be noted that, in cultured monocytes-macrophages, inflammatory mediators stimulated mainly the synthesis of PAI-2 (Figure 3).^16^ Studies in non-human primates and in healthy volunteers receiving low-dose endotoxin or TNF have shown a sudden increase in plasma t-PA levels, indicative of EC activation, which coincided with the activation of the fibrinolytic system, assessed by augmented plasmin-α_2_-plasmin inhibitor complexes. However, the fibrinolytic activity was rapidly offset by the subsequent and long-lasting increase in the plasma levels of PAI-1.^11^,^33^ A marked increase in plasma PAI-1 during endotoxemia has consistently been found in every animal species.^33^,^103^ Studies at tissue level in animals challenged with endotoxin or TNF showed a strong elevation of PAI-1 mRNA in tissues, including those most commonly affected by microthrombi during sepsis (kidney, adrenals, lung and liver). PAI-1 expression (RNA, protein or activity) was detected primarily in ECs at all levels of the vasculature (arteries, veins and capillaries).^33^ The up-regulation of PAI-1 synthesis in ECs of multiple tissues clearly suggests that plasma PAI-1 may originate from these cells during endotoxemia. In this respect, a possible contribution of platelets seems unlikely because neither thrombocytopenia nor antiplatelet agents affected the increase in plasma PAI-1 in rat endotoxemia.^33^

Endotoxin administration also causes changes in t-PA and u-PA expression in rats and mice. In both animal species, an increase in t-PA mRNA was observed in different tissues.^33^ However, this increase was not associated with increased t-PA activity, because of the very large amounts of PAI-1 produced in the same tissues. u-PA mRNA behaved differently in rats and mice, being increased in rat kidney^104^ but markedly decreased in murine tissues, particularly in adrenals and kidneys (tubular epithelial cells).^105^ Interestingly, the decrease in uPA expression correlated with fibrin deposition in kidneys and adrenals of endotoxin-treated mice.^106^ In rabbits given a continuous infusion of endotoxin or TNF, a marked reduction in fibrinolytic activity was observed in glomeruli own to a reduced production of PAs.^107^ Altogether these data indicate that suppression of the fibrinolytic system through increased PAI-1, mostly mediated by ECs, and other tissue- and species-specific alterations, such as decreased u-PA in the mouse model, are essential for fibrin deposition in tissue-specific vasculature, at least in experimental models of sepsis. This concept is strongly supported by experiments in mice with targeted disruptions of genes encoding fibrinolytic proteins. Mice with a deficiency of plasminogen activators have more extensive fibrin deposition in organs when challenged with endotoxin, whereas PAI-1 knockout mice, in contrast to wild-type controls, have no microvascular thrombosis upon endotoxin challenge.^106^,^108^

Local changes in fibrinolysis, however, may involve also other mechanisms. Administration of low-dose endotoxin to chimpanzees caused a marked depression of fibrinolytic activity in the bronchoalveolar fluid, due to increased levels of PAIs, especially PAI-2.^109^ In this case, macrophages rather than ECs were more likely responsible for down-regulating fibrinolysis. Similarly, in a mouse model of hypoxia, a clear increase in PAI-1 mRNA in the lung tissue was observed, which was associated with a decrease in both t-PA and u-PA mRNAs.^33^,^108^ These local changes were shown to play an important role in hypoxia-induced vascular fibrin deposition in the lung. Again, immunolocalization studies identified macrophages as the predominant source of PAI-1 within the hypoxic lung. Therefore, the cellular origin of fibrinolytic components may be quite different, depending on the tissue, the animal species, and the pathological condition.

In human sepsis-associated DIC, studies of endothelial fibrinolysis-related properties at tissue level are scarce. In patients with adult respiratory distress syndrome (ARDS) secondary to sepsis, the fibrinolytic activity of bronchoalveolar fluid was markedly lower than that of patients with interstitial lung disease or normal subjects, due to the appearance of PAI activity.^33^,^110^ In situ hybridization on lung biopsies showed that, like in animal models, macrophages rather than ECs were the main source of PAI-1. The majority of studies in patients with sepsis has been restricted to circulating fibrinolytic markers. In septic patients a sustained increase in plasma PAI-1 has been consistently reported by numerous investigators^33^,^103^,^111^,^112^ and, in some studies, PAI-1 turned out to be a prognostic marker in patients with septic shock.^33^,^111^,^112^ Plasma t-PA antigen was also found to be elevated in septic patients,^33^ but the net effect of the changes in t-PA and PAI-1 was definitely antifibrinolytic. That impaired fibrinolysis is an important factor in the pathophysiology of human sepsis is supported by the finding that a 4G/5G polymorphism in the PAI-1 promoter, leading to high PAI-1 expression, was associated with poor outcome in meningococcal sepsis^11^,^33^ and with multiple organ dysfunction and shock in pneumonia-induced severe sepsis.^113^

During the last decade evidence has accumulated that other, thrombin-dependent mechanisms may contribute to the impairment of fibrinolysis during sepsis. Besides stimulating the synthesis and release of fibrinolytic components in different cells, thrombin is able to affect fibrinolysis in different ways: a) it stabilizes the clot by activating factor XIII, which introduces covalent bonds between fibrin strands and incorporates α_2_-plasmin inhibitor into the clot;^114^ b) it causes the formation of more compact and less permeable clots in a concentration-dependent way;^115^ and c) it reduces plasmin generation via activation of thrombin-activatable fibrinolysis inhibitor (TAFI) (procarboxypeptidase U), a plasma procarboxypeptidase that, once converted into the active enzyme, TAFIa, removes the C-terminal lysines from partially degraded fibrin, thereby reducing the binding of t-PA and plasminogen to the clot.^116^ All of these mechanisms have been associated with resistance to fibrinolysis.

In sepsis, enhanced thrombin generation induced by activated cells might well contribute to inhibition of fibrin removal by the fibrinolytic system both by modifying structure and stability of the fibrin network and by activating TAFI. To our knowledge there are no available ex vivo studies on the characteristics of fibrin deposited in tissues during sepsis. Recently, however, Campbell et al.^117^ reported that ECs stimulated by inflammatory cytokines to express TF, similarly to normal TF-expressing extravascular cells (fibroblasts, SMCs), caused the production of abnormally dense fibrin networks that resisted fibrinolysis. Moreover, activated platelets, commonly found in sepsis, not only alter the fibrin structure and reduce susceptibility to lysis via the direct interaction between fibrin and αIIbβ3 integrin^117^ but also release, together with the causative micro-organism, inorganic polyphosphates, which have been recently shown to modify the fibrin architecture and to reduce the binding of t-PA and plasminogen to fibrin, thereby increasing fibrin resistance to fibrinolysis.^118^

With respect to TAFI, evidence is accumulating that it might be importantly involved in the sepsis-associated suppression of fibrinolysis. In vitro, LPS-stimulated human monocytes inhibit fibrinolysis through a tissue factor-mediated enhancement of TAFI activation and make clots resistant to the profibrinolytic activity of heparins.^119^ Because TF-expressing monocytes-macrophages drive blood clotting activation in sepsis, our findings provide an additional mechanism whereby these cells may favor fibrin accumulation in the microcirculation. Experiments in mice with targeted disruption of the TAFI gene did not allow clear-cut conclusions about the role of this protein in endotoxemia.^120^ However, in a rat model of polymicrobial sepsis, a significant reduction of TAFI levels was observed,^121^ probably due to TAFI consumption. Moreover, in rat endotoxin-induced DIC, it was demonstrated that blocking TAFIa with a synthetic inhibitor (BX528) attenuated fibrin resistance to endogenous fibrinolysis.^122^ More recently, Muto et al.,^123^ using a rat endotoxemia model and a rat sepsis model induced by *Pseudomonas aeruginosa*, showed that the administration of another synthetic TAFIa inhibitor (EF6265) 1 and 2 hours after the intravenous injection of LPS and bacteria, respectively, resulted in decreased fibrin deposition in the kidney and liver without significant changes in platelet count and plasma fibrinogen levels. Notably, this compound also significantly decreased laboratory markers of organ dysfunction in both models and the inflammatory response in the bacterial infection model. In a baboon model of *Escherichia coli*-induced sepsis, Binette et al.^124^ demonstrated that TAFI is rapidly activated and cleared and that a monoclonal antibody specifically inhibiting thrombin-TM-dependent TAFI activation prevented the TAFI activation/consumption and enhanced the rate of fibrin degradation. Therefore, in an in vivo model in which multiple potential activators of TAFI are present (thrombin, plasmin, elastase), thrombin-TM appeared to be the predominant activator and the generated TAFIa down-regulated fibrinolytic activity. In human studies, TAFI levels (activity and/or antigen) were found consistently decreased in septic patients as well as in healthy volunteers with low-grade endotoxemia.^125^–^127^ It was unclear whether activation or consumption or both were the cause of this decrease. In a comprehensive study evaluating TAFI activation markers on a rather large series of patients with severe meningococcal infection, Emonts et al.^128^ demonstrated that TAFI levels were significantly decreased at admission compared to the convalescence state and were lower in patients with septic shock. More important, the levels of TAFI activation peptide (TAFI-AP) were augmented in patients with DIC as compared with those without. In addition both TAFI-AP and inactivated TAFIa (TAFIai), another marker of TAFI activation, were significantly higher in non-survivors versus survivors and strongly correlated with severity scores of the disease. It thus appears that TAFI activation does occur in severe sepsis and that the measurement of TAFI activation markers may be clinically useful. The pathophysiological and clinical relevance of TAFI is further supported by the observation that a functional single nucleotide polymorphism in the TAFI gene that leads to the substitution Thr325Ile and produces increased TAFIa stability and activity was associated with a poor outcome of meningococcal sepsis.^129^

### Role of endogenous inflammatory mediators in coagulation/fibrinolysis changes:

While in vitro the pathogen, its derivatives and the main inflammatory mediators are able to influence to variable extent each of the mechanisms described above, their role during experimental and human sepsis or endotoxemia is more difficult to establish. With respect to the up-regulation of procoagulant pathways, various inflammatory mediators are likely involved, besides the micro-organism itself and the products it expresses (for instance endotoxin). The main cytokines, i.e. TNF, IL-1 and IL-6, can activate blood coagulation in humans and primates.^40^ This occurs most probably via the TF pathway, although direct, definitive proof for induction of TF synthesis by these cytokines in vivo is very limited. As to the relative contribution of individual cytokines, neutralization studies with specific antibodies would suggest a major role of endogenous IL-6 and, to a lesser extent, of IL-1.^40^ Although TNF can activate coagulation when injected in animals or in man and can induce TF in vitro, endogenous TNF does not appear to play a significant role in activation of coagulation during endotoxemia or gram-negative sepsis. Knowledge of the in vivo effects of cytokines on anticoagulant pathways is rather scarce. A neutralizing anti-TNF antibody attenuated the release of soluble TM into the circulation of baboons with *E. Coli*-induced severe sepsis.^40^ Moreover, both TNF and IL-1, when administered to mice, reduced protein C mRNA expression in liver and other organs.^40^ As to the fibrinolysis, the neutralization of endogenous TNF activity by various means in humans or chimpanzees almost completely prevented endotoxin-induced fibrinolytic changes, including both t-PA and PAI-1 release.^40^ Inhibition of TNF has also been shown to down-regulate plasma PAI-1 levels in a clinical setting other than sepsis.^130^ Similar effects were obtained in baboons with severe sepsis following the administration of recombinant IL-1 receptor antagonist.^40^ In mice challenged with endotoxin, anti-TNF treatment attenuated plasma PAI-1 increase and reduced PAI-1 mRNA expression in the liver.^40^ IL-1 and especially TNF, therefore, appear to be strongly involved in PAI-1-mediated suppression of fibrinolysis associated with sepsis. In a very recent comprehensive study using a baboon model of *E. coli*-induced severe sepsis, Silasi-Mansat et al.^131^ demonstrated that treatment with compstatin, a potent C3 convertase inhibitor, effectively attenuated the inflammatory response and the associated DIC, and improved the function of several organs (heart, kidney, liver). Compstatin decreased TF expression in lungs and other organs and this decrease correlated with the observed reduction in monocyte-macrophage infiltration. The reduced TF levels were accompanied by a reduced PAI-1 expression and by a protection against TFPI and TM down-regulation in ECs. It thus appears that complement-derived mediators play an important role in the up-regulation of cell TF and PAI-1, and in the down-regulation of anticoagulant pathways during sepsis. Altogether, these findings clearly show that, as predicted by in vitro studies, inflammatory stimuli are indeed able to elicit the main mechanisms responsible for in vivo thrombin generation and fibrin deposition during sepsis.

## Pathogenetic mechanisms of multiple organ dysfunction syndrome (MODS)

MODS is the hallmark of severe sepsis and septic shock and represents the main cause of the high mortality in these conditions. Various closely interlinked mechanisms have been proposed to explain this dramatic event, all of which eventually result from the global hyper-inflammatory response to the triggering pathogen.^132^ In the early phase, overproduction of numerous cell-derived (mainly cytokines) and soluble (e.g. complement activation products) inflammatory mediators causes widespread activation/dysfunction and injury of ECs resulting in increased vascular permeability, prothrombotic cell surface changes, up-regulation of adhesion molecules with subsequent recruitment and extravasation of polymorphonuclear and mononuclear cells.^132^,^133^ Although endothelial activation/dysfunction is generalized, the local susceptibility to endothelial changes can vary widely because of the heterogeneity of EC properties between vascular beds in different organs.^133^ In the later phase of MODS, the recruited cells, together with other tissue cells, such as mast cells, release and produce a number of mediators, including cytokines, thus amplifying inflammation in the interstitial space.^132^ The ensuing parenchimal cell injury and organ dysfunction is most likely due to several simultaneously acting mechanisms. A critical role has been attributed to neutrophils owing to their inappropriate positioning within the microvasculature (because of endothelial dysfunction) and to their massive activation with subsequent release of reactive oxygen and nitrogen species and a mixture of lytic enzymes, besides many other factors.^77^ High concentrations of selected cytokines in the interstitial space might be directly toxic to vulnerable parenchyma and play a role particularly in MODS with severe leukopenia.^132^ The complex interplay between inflammation and the haemostatic system during sepsis frequently leads to DIC, which causes massive fibrin formation and its persistent deposition in the microcirculation. Several lines of evidence support an important role of DIC in MODS.^11^ Numerous histological studies of tissues from septic patients and from animals with bacteremia or endotoxemia have, indeed, demonstrated the presence of thrombi in small and mid-size vessels of multiple organs. These intravascular thrombi appear to be clearly related to organ ischemia-necrosis and dysfunction. Moreover, in experimental studies, amelioration of DIC by various interventions improves organ failure and, at least in some cases, mortality. In some studies, the attenuation of systemic activation of intravascular coagulation was clearly associated with removal of locally deposited fibrin which may, at least in part, contribute to the improvement of organ function. Finally, DIC has been shown to be an independent predictor of organ dysfunction and mortality in patients with sepsis.

Recent evidence indicates the existence of new players in sepsis-associated organ dysfunction and failure, namely extracellular histones, that originate mainly from apoptotic or necrotic cells and therefore can be considered as late mediators of MODS. Histones are normal nuclear proteins that serve an essential architectural role by forming the core around which DNA is wrapped in eukaryote cells and contribute to gene regulation. These proteins can also be found in the extracellular environment where they kill invading pathogens thus possibly participating in the innate immune protection. Recently Xu et al.^14^ have shown that histones are major mediators of injury during sepsis. In vitro they were toxic for human endothelial cells and this toxicity was mainly due to the histones H3 and H4. When injected into mice, histones mimicked the symptoms of sepsis, including microvascular thrombosis, organ failure and death. More importantly, antibodies against histone H4 protected mice in different models of endotoxemia and sepsis. A remarkable finding of the study is that recombinant APC was able to degrade histones both in vitro and in vivo thus lowering the histone toxicity towards ECs in vitro and preventing lethality in histone-treated animals. Of note, histones and their APC-induced degraded forms were detected in plasma from septic animals and patients. Therefore histones add to the list of APC substrates that make this protease effective against sepsis. Interestingly, histones are not the only nuclear proteins involved in sepsis. HMGB1 is a nuclear DNA-binding protein involved in nucleosome stabilization and gene transcription. However, when released into the extracellular environment, it becomes a lethal mediator of systemic inflammation.^134^ HMGB1 increases in the circulation in different animal models of sepsis as well as in septic patients. When injected in mice, HMGB1 is lethal and a recent study showed that it promotes the development of microvascular thrombosis in rats likely by stimulating TF expression in monocytes and by reducing the activity of thrombin-TM complex thereby inhibiting protein C activation.^39^ Passive immunization with anti-HMGB1 antibody confers significant protection against lethal endotoxemia, sepsis and LPS-induced lung injury, even when antibody administration is delayed.^134^ These observations raise the possibility that nuclear contents in circulation might indicate severe damage and contribute to host mortality. While HMGB1 is known to be released actively by innate immune cells (monocytes-macrophages) stimulated by numerous inflammatory mediators (including cytokines) and passively by necrotic cells, it is not yet clear where histones come from during sepsis. Neutrophils are an attractive possibility, as they are the most abundant white blood cells participating in severe sepsis.^15^ Furthermore, platelets activate neutrophils to make neutrophil extracellular traps (NETs) during sepsis.^135^ NETs are made of chromatin, that is DNA and core histones. Although they might help killing micro-organisms during sepsis, they may also lead to tissue damage and organ failure. A major source of histones is most probably massive cell apoptosis or necrosis overwhelming the clearance capacity of mononuclear phagocytes, thereby permitting histones to enter into circulation. Altogether, these results clearly demonstrate that histones are toxic to endothelium, are released during sepsis and are important mediators of sepsis with devastating effects.

## Concluding remarks

Considerable progress has been made in our knowledge on the mechanisms underlying sepsis-associated DIC and MODS. Although direct interactions between the infectious agent and the haemostatic system do occur, the numerous inflammatory mediators generated by the host in response to the causative micro-organism, particularly cytokines and complement activation products, are now believed to play a pivotal role in driving the major changes responsible for massive thrombin formation and fibrin deposition, namely the aberrant expression of the coagulation trigger TF by different cell types (especially monocytes-macrophages) and the impairment of both physiological anticoagulant mechanisms (antithrombin, protein C pathway and TFPI) and fibrinolysis, mainly due to inflammation-induced endothelial dysfunction. The ensuing thrombosis in the microcirculation and ischemia are generally thought to contribute to parenchimal cell injury/death and single or multiple organ dysfunction, together with other rather well-established mechanisms, also elicited by the overwhelming inflammatory response, such as neutrophil- and macrophage-derived factors and selected cytokines with direct toxic effects. The recently published evidence that extracellular histones, particularly H3 and H4, “are major mediators of death in sepsis”^14^ add to our understanding of the MODS pathogenesis. These nuclear proteins, together with others such as HMGB1, are released into the circulation mainly by apoptotic or necrotic cells and thus may represent late mediators that amplify inflammation, coagulation, cell death and organ failure.

Whether the present knowledge on the pathogenesis of sepsis-associated DIC and MODS will prove useful for diagnostic and therapeutic purposes remains to be established. The diagnosis of DIC is still based on the combination of a typical underlying disease, such as sepsis, with laboratory markers, including platelet count, prothrombin time, activated partial thromboplastin time, fibrinogen concentration, and a fibrin-related marker, reflecting intravascular fibrin formation, such as D-dimer, all of which are used in the DIC scores.^11^ However, new parameters are being investigated that could be of clinical utility. For instance, HMGB1 is persistently augmented in septic patients, being significantly higher in non survivors than in survivors and the plasma concentration of this protein has been proposed as possible prognostic markers of DIC and organ failure.^136^ Moreover, elevated plasma levels of nucleosomes have been reported in septic patients that were proportional to the disease severity (highest values in septic shock)^137^,^138^ and single histones H3 and H4 were increased in patients with severe sepsis.^14^ Nuclear proteins, therefore, might be new sensitive biomarkers of disease progression and useful predictors of outcome.

As regards the supportive treatment for sepsis-associated haemostatic abnormalities, different strategies have been developed based on the insights into the pathogenetic mechanisms responsible for thrombin formation and fibrin deposition. However, the use of TF inhibitors, which would be the most logical treatment considering the widely accepted pivotal role of TF in blood clotting activation during sepsis, is still a matter of debate. A phase III trial with recombinant TFPI did not show a overall survival benefit in septic patients.^139^ Likewise, treatment with antithrombin concentrates to restore one of the most important anticoagulant mechanisms shown to be impaired in sepsis, despite some reported beneficial effects (improvement of laboratory parameters, shortening of DIC duration, amelioration of organ function), in a large-scale clinical trial failed to significantly reduce the mortality of septic patients.^140^ So far, the only anticoagulant drug showing the expected promising results is recombinant human APC, which has been evaluated in several clinical trials and is indicated for the reduction of mortality in adult patients with severe sepsis at high risk of death.^1^ The beneficial effects of this drug have been attributed not only to the restoration of the protein C anticoagulant pathway, which is strongly impaired in septic patients, but also to its well-known anti-inflammatory action.^84^,^98^ The finding that APC degrades histones^14^ add a new mechanism whereby the drug exerts it beneficial effects especially in severe sepsis. Because histones seem to be critical mediators of organ dysfunction and death in septic patients, an attractive approach to treat MODS and prevent death could be the development of effective histone antagonists which might prove therapeutic without the bleeding complications that can result from APC therapy.

Finally, the recent evidence that complement-coagulation interplay strongly contributes to the progression of severe sepsis^131^ suggests that blocking the harmful effects of complement activation products, especially during the organ failure stage of severe sepsis, is yet another potentially important therapeutic strategy.

## Figures and Tables

**Table 1:** Inflammatory stimuli and conditions inducing tissue factor synthesis/expression in endothelial cells and/or mononuclear phagocytes.

**Micro-organisms and derivatives** Whole bacteria Rickettsiae and viruses Lipopolysaccharides (LPS) Peptidoglycan and lipotheicoic acid Staphylococcal and streptococcal exotoxins Shiga toxin (verotoxin-1) H. pylori-neutrophil activating protein **Complement activation products** C5a C5b-9 **Antibodies and immune complexes** Bradykinin Cytokines Tumor necrosis factor-α (TNF-α) Interleukins (IL-1, IL-6) Interferon γ	**Chemokines** Monocyte chemoattractant protein-1 (MCP-1) Interleukin-8 Leukotactin-1 **Acute phase proteins** C reactive protein (CRP), Long pentraxin α_1_-acid glycoprotein **Growth factors (GF)** Vascular endothelial GF (VEGF) Platelet-derived GF (PDGF) Monocyte colony-stimulating factor (M-CSF) GM-CSF **Cell-cell interactions** Clotting proteins (thrombin, factor Xa, fibrin) **Hypoxia** High Mobility Group Box-1 (HMGB-1)
